# Dose-dependent effect of caffeine supplementation on judo-specific performance and training activity: a randomized placebo-controlled crossover trial

**DOI:** 10.1186/s12970-019-0305-8

**Published:** 2019-09-05

**Authors:** Krzysztof Durkalec-Michalski, Paulina M. Nowaczyk, Natalia Główka, Aleksandra Grygiel

**Affiliations:** 1 Institute of Human Nutrition and Dietetics, Poznań University of Life Sciences, Wojska Polskiego 31, 60-624 Poznań, Poland; 2 Department of Food and Nutrition, Poznan University of Physical Education, Królowej Jadwigi 27/39, 61-871 Poznań, Poland

**Keywords:** Caffeine, Combat sports, Judo, Physical performance, *Special Judo Fitness Test*

## Abstract

**Background:**

Caffeine (CAF) supplementation could have a positive impact on physical performance and sport abilities. Nevertheless, the CAF-induced, dose-dependent influence on discipline-specific performance and combat activity in combat sports have not been sufficiently investigated. The aim of this study was to examine the effect of single ingestion of 3, 6, or 9 mg/kg body weight of CAF and placebo (PLA) on judo-specific performance and sparring combat activities.

**Methods:**

In a randomised double-blind placebo-controlled cross-over design, acute pre-exercise supplementation with CAF (3, 6, or 9 mg/kg body weight) and placebo PLA in 22 male highly-trained judoists was examined. The study protocol involved five separate testing sessions using the *Special Judo Fitness Test* (SJFT) with heart rate monitoring, three judo sparring combats and evaluation of the rate of perceived exertion (RPE) using the Borg scale.

**Results:**

Six and 9 mg/kg CAF improved SJFT performance, while 9 mg/kg increased combat activity. Three mg/kg CAF lacked any apparent positive ergogenic effect. Among athletes, who include CAF-containing products in their habitual diet (consumers), only 9 mg/kg CAF effectively enhanced SJFT performance, while in those who do not consume CAF-containing products at regular basis (non-consumers), the enhancing effect was achieved even at 6 mg/kg.

**Conclusions:**

Regarding combat sports, higher (6–9 mg/kg) than currently recommended CAF dosages (3–6 mg/kg) are apparently more effective in terms of judo-specific performance. However, the ergogenic CAF effect is not only dose-dependent, but it is also related to customary CAF consumption.

**Trial registration:**

Clinical Trials Gov, NCT03822663. Registered 28 January 2019 **-** Retrospectively registered

**Electronic supplementary material:**

The online version of this article (10.1186/s12970-019-0305-8) contains supplementary material, which is available to authorized users.

## Background

The use of dietary supplements is highly prevalent in athletes, mainly in order to enhance physical capacity, exercise performance and achieve other ergogenic benefits. These actions stimulate adaptation and recovery or support training/competition abilities [1]. According to the *International Olympic Committee* consensus statement [1], there are few supplements with an adequate level of evidence-based support to suggest that they have a positive impact on physical performance and sport abilities. One of the most researched ergogenic supplements is caffeine (CAF) [1].

CAF (1,3,7-trimethylxanthine) is a stimulant found in coffee, tea, energy drinks, chocolate and supplements like guarana, kola and bissey nut [2]. CAF is absorbed through the gastrointestinal tract and metabolised by the liver [3]. About 15–45 min after ingestion, blood CAF concentration rises, with its peak observed after (on average) 60 min (15–120 min) [3, 4]. The half-life for CAF elimination ranges between 2.5–10 h for doses lower than 10 mg/kg [3]. Several studies proposed multiple mechanisms to explain CAF supplementation effects on sport performance [5–12], including its action as an adenosine receptor antagonist [8, 13, 14], central nervous system (CNS) activity modulation, muscle excitation-contraction coupling [13, 15] and motor unit recruitment [4, 16]. It can also reduce fatigue-induced psycho-physical symptoms [8, 17–19]. By elevating blood catecholamine concentration [4, 20, 21], CAF increases glycolytic activity and enhances muscle energy production capacity [4, 22, 23]. The *International Society of Sports Nutrition* proposes 3–6 mg/kg CAF consumption 15–30 min prior to exercise. Doses > 9 mg/kg may not confer additional benefits [5].

All of the mentioned CAF-related improvements appear to be valuable in combat sports where efforts are characterised by intermittent high-intensity exercises [4, 5]. Among combat sports judo is an esteemed Olympic discipline. For judoists, it is crucial to achieve high physical abilities like performance, power and muscle strength, agility and endurance while maintaining the proper weight and/or optimal exercise capacity if pre-competition body mass must be urgently reduced [24–26]. Further, in judo, short bouts of intensive, powerful and dynamic actions, interspersed with actions of low intensity, require impressive technical and tactical skills [4]. Customarily, judoists perform 5–7 matches during international competitions, each of them lasting up to 4 min, in accordance with current rules [27]. During each match an athlete performs numerous actions (attacks and/or defenses). Short bursts of activity are energetically covered primarily through anaerobic metabolism, but the maintenance of the intermittent work during the entire competition and effective recovery between matches requires high aerobic metabolism capacity [24]. Taking into account the characteristic of efforts in judo, the required technical and tactic abilities, and the exposition for physical and psychological conditions during the competition, caffeine with its mechanisms of action and ergogenic properties seems to be a promising supplement for enhancing performance in judo.

However, there are hardly any data on the individual CAF-induced and dose-dependent changes in discipline-specific performance in combat sports. According to our scientific and practical observations, we hypothesised that there would be a dose-dependent relationship between CAF supplementation and performance. At a higher dose, the athletic benefits would increase, which could be affected by the habitual use of CAF. Therefore, this study aimed to examine the effect of acute CAF ingestion (3, 6 or 9 mg/kg body weight) on judo-specific exercise performance and combat activities.

## Methods

### Participants

**Fig. 1:**
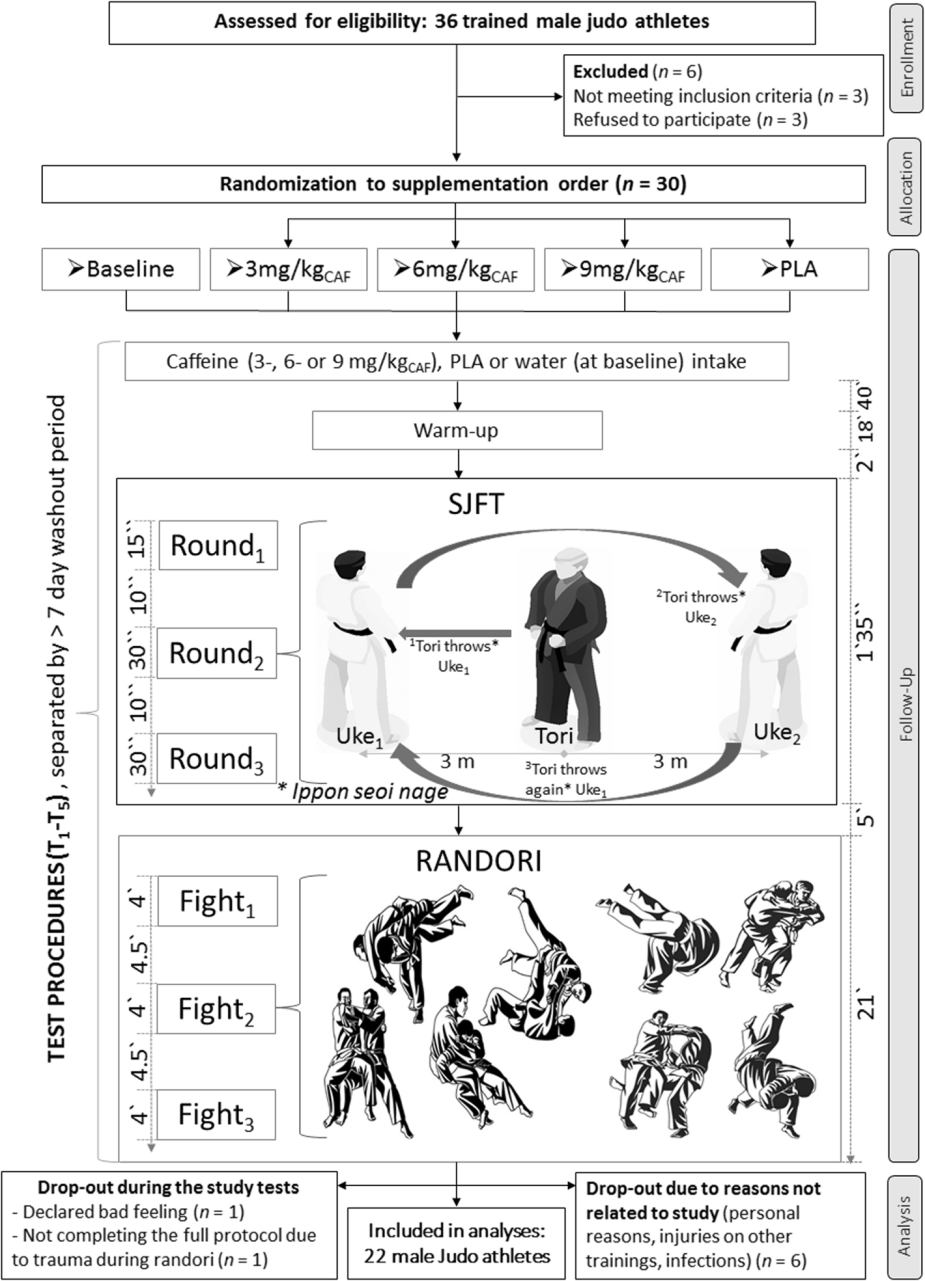
A flow chart of the study design. Abbreviations: SJFT, *Special Judo Fitness Test*; T_1_, 1st series of test procedures; T_5_, 5th series of test procedures; *Tori*, the studied judoka who performs a SJFT throw test; PLA, placebo; *Uke*, partners (in the same weight category and of similar height) thrown by *Tori*

Thirty trained male judoists were initially enrolled in this study. However, 22 judoists completed the study and were included in analyses (Fig. 1; Table 1). The athletes were members of the Polish Judo Clubs from Wrocław, Poznań and Jarocin. They occupied leading positions in national competitions and also represented Poland in international competitions.

**Table 1:** Anthropometric characteristics and training experience of male judo athletes

Indicator	All	Caffeine consumers	Caffeine non-consumers
	*n* = 22	*n* = 10	*n* = 12
Age (years)	21.7 (3.7)	22.8 (4.8)	20.8 (2.4)
Body height (cm)	178 (7)	177 (8)	179 (7)
Body weight (kg)	76.4 (11.1)	76.6 (10.1)	76.2 (12.4)
BMI (kg/m^2^)	24.0 (2.1)	24.4 (1.8)	23.7 (2.3)
Training experience (years)	11.0 (4.5)	10.9 (4.7)	11.2 (4.6)

Values are expressed as means (± SD)

The primary recruitment strategy was to contact the coaches responsible for training judo athletes. They enabled the identification, inclusion and confirmation of the inclusion criteria aspects declared by the participants. They also supported training control and supplementation compliance. The inclusion criteria were: good health, a valid and up-to-date medical certificate that confirmed the athlete’s ability to practice sports, at least 4 years of training experience and participation in a minimum of four judo workout sessions a week. The exclusion criteria were: current injury, any health-related contraindication, declared general feeling of being unwell and unwillingness to follow the study protocol. The drop-outs were predominantly independent from the study protocol (Fig. 1). The reasons for drop outs were: personal, infections and/or minor injuries during customary training. The entire study protocol for each participant lasted 5 weeks. All athletes declared that before and during the study protocol they did not introduce any changes in their lifestyles, training elements, nutrition or supplementation, and that they had not been using any medications and/or supplements with potential ergogenic effects, other than those supplied by the authors of this study. For this study, athletes were also divided depending on daily regular caffeine consumption into - “caffeine consumers” - athletes who consumed more than 160 mg of CAF each day, and “caffeine non-consumers” - those who consumed less than 160 mg of CAF on a daily basis or consumed products containing CAF less frequently. The amount of 160 mg of CAF refers to the average amount of CAF found in two cups (240 ml per cap) of coffee [28]. During the study, the participants were in the usual and non-starting training period in which they did not regulate body weight or did not make any specific changes to training procedures. In accordance with the 1975 Declaration of Helsinki, all participants consented to participate in the research procedures before the study began. This study was approved by the Bioethics Committee at Poznan University of Medical Sciences and was registered at ClinicalTrials.gov (NCT03822663). The study was registered retrospectively, since registration was not required when study enrolment started. The authors confirm that all ongoing and related trials associated with this intervention are registered. The trial was conducted from May to June 2017 and May to June 2018. It complies with the CONSORT statement for randomised trials, as shown in Fig. 1 and Additional file 1: Table S1.

G*Power software (version 3.1.9.4, Universität Düsseldorf, Germany) was used to calculate sample size required to obtain a power of approximately 80% (α = 0.05) and large effect size partial eta squared 0.14 in analysis of variance (ANOVA) with repeated measurements (RM) within factors. Analysis indicated that a sample size of 9 would be suitable for detecting a difference between five measurements. In one previous study, a sample of 18 was appropriate to detect significant differences in SJFT results after acute CAF supplementation in young judoists [29].

### Study design and protocol

The study protocol consisted of acute supplementation with three doses of CAF or PLA in a randomised double-blind placebo-controlled crossover design. The primary outcomes were changes in judo-specific exercise performance and subsequent combat activities. Between treatments, a 7-day washout period was introduced. This period was likely sufficient given the kinetics of CAF excretion from the body [3]. The participants were intimated on the testing procedures, protocols and equipment before study commencement.

The participants were first enrolled by the authors and then randomly assigned (stratified randomisation) to the supplementation groups with specific codes by an impartial biostatistician. The main study protocol involved five separate visits (T_1_–T_5_) and included exercise tests (SJFT and judo sparring combats (*Randori*)) conducted in natural conditions at the judo training centres. The participants performed exercise tests before (baseline) and after each treatment (CAF or PLA). All testing was performed at the same time of day (afternoon hours). Moreover, 3 hours before the exercise tests, participants consumed standardised small meals. They were also instructed to avoid strenuous exercise for the 24 h preceding each test session.

### Supplementation

The experimental procedure for each athlete included an acute 3, 6 or 9 mg/kg body weight CAF supplementation and PLA treatment in a crossover regimen. CAF (pure pharmaceutical caffeine, KFD Nutrition, Poland) and PLA (maltodextrin) were administered dissolved in 350 mL water. On testing days, the supplements were taken 60 min before the exercise capacity test session (Fig. 1). The preparations were administrated to each participant in containers marked with a unique code. In accordance with the recommended blind procedure, the preparations were made in advance (after morning anthropometric measurements) by the research team member who did not directly participate in the investigations. Regarding double blinding, neither the researchers nor the participants knew whether CAF or PLA was administered. Only the head of the department had access to the randomisation information, which was only revealed after protocol cessation. The preparations were administered at a strictly specified time before the exercise tests, and consumption compliance was controlled by the investigators along with the trainers of the studied athletes.

### Anthropometric measurements

Anthropometric measurements were taken with the participants in a fasted state during the morning hours at each visit. Body mass and height were measured using a professional medical scale with a stadiometer (WPT-60/150OW, RADWAG®, Poland). Further, the proper hydration level was verified via urine specific gravity measurement, with URYXXON® Relax (Macherey-Nagel, Germany), and results < 1.020 indicated proper hydration.

### Exercise tests

During each exercise session (T_1_–T_5_), all athletes performed: *1)* a standardised 18-min warm-up; *2)* one SJFT and *3)* three sparing combats (Fig. 1). All test procedures (SJFT and *Randori* combats), were evaluated in real time by two independent coaches. Additionally, they were visually registered, and the final evaluation and verification was done by a member of the research team (judo black belt coach). This triple control allowed precise counting of all technically well-performed throws and attacks. At the end of each testing session (4.5 min after the final combat), the athletes were asked to rate their perceived exertion (RPE) using the Borg scale (9–20) according to published recommendations [30]. RPE was evaluated at the end of testing session to minimise effects of data collection on exercise performance.

#### SJFT

The classical SJFT was performed in accordance with all the recommendations described previously [31–33]. SJFT consists of 3 consecutive rounds (1 × 15 s, and 2 × 30 s with 10-s recovery intervals between them) of “*Ippon-seoi-nage*” throws performed by the studied judoka (*Tori*) on two partners (*Uke*) (Fig. 1). The main goal of the subject is to perform as many throws as possible; the athlete’s performance is evaluated by assessing the total number of throws (TOT_throws_) completed during the SJFT and during each separate round. Further, *Tori* heart rate was continuously monitored during exercise and registered immediately after (HR_RA_) and 1 min after (HR_1minAF_) the SJFT bout using a telemetric system (Polar, Finland), and an SJFT_Index_ ((HR_RA_ + HR_1minAF_)/TOT_throws_) was calculated [25, 31–33]. The judoists were familiar with the SJFT because of previous research and trainings.

#### Combat activity

During *Randori*, the judoists performed three 4-min judo matches [27] separated by 4.5-min rest intervals. During these fights studied athletes competed with opponents in the same weight category and with a similar sport level relative to his ranking. If the fight was won by *ippon*, it was not interrupted but was continued to maintain the repetition of the fight duration. During combat activity analysis, only actual (not feigned) attacks performed in the standing position were considered. The judoists were obliged to fight in the usual style focused on actual effectiveness and final winning with an opponent (on points or by *ippon*).

### Statistical analysis

All variables were checked for normal distribution using the Shapiro-Wilk test. For variables with normal data distribution, the effects of CAF dosage or PLA on SJFT and fight performance were tested by ANOVA with RM. Two-way ANOVA with RM was performed to evaluate the effect of habitual CAF consumption on supplementation effect (CAF dosage x customary CAF consumption). A Huynh-Feldt adjustment was made when sphericity was violated (as indicated by Mauchly’s test). Post-hoc comparisons were performed by Fisher’s least significant difference (LSD) test. Effect sizes were calculated using partial eta squared (${\eta}_p^2$; 0.01 – small effect, 0.06 – medium effect and 0.14 – large effect [34–36]). For variables not normally distributed, Friedman’s ANOVA, followed by pairwise Wilcoxon signed-rank test, were applied. Effect sizes were calculated using Kendall’s concordance coefficient *W* (0 – no agreement and 1 – complete agreement). The same approach was implemented to verify the effect of the number of attacks in *Randori* combat outcomes within particular CAF doses. Differences between SJFT_R2_ and SJFT_R3_ outcomes were tested with a T test for dependent variables or pairwise Wilcoxon signed-rank test, with effect size expressed as Cohen’s *d* or rank correlation coefficient *r*_*c*_. With regard to variables not normally distributed, the effect of habitual CAF consumption was verified by dividing the entire studied group into “consumers” and “non-consumers” subgroups. Further analyses in these two subgroups were performed according to above described scheme, starting from checking the data distribution. Moreover, the statistical analysis was complemented by comparing all the variables between CAF ‘consumers’ and ‘non-consumers’, via a T test for independent variables (for normally distributed data; effect size expressed as Cohen’s *d;* 0.20 – small effect, 0.50 – medium effect and 0.80 – large effect [34]) or the Mann-Whitney U test (variables not normally distributed; effect size expressed as Glass’s rank-biserial correlation coefficient (*r*_*g*_); interpretation according to correlation coefficient). HR (robust to violations of normality) and RPE (ordinal scale) results were analyzed with parametric and non-parametric test, respectively, regardless of data distribution. Statistical significance was set at *P* <  0.05, and data were analysed using the STATISTICA-13.3 software program (StatSoft Inc., USA).

## Results

**Fig. 2:**
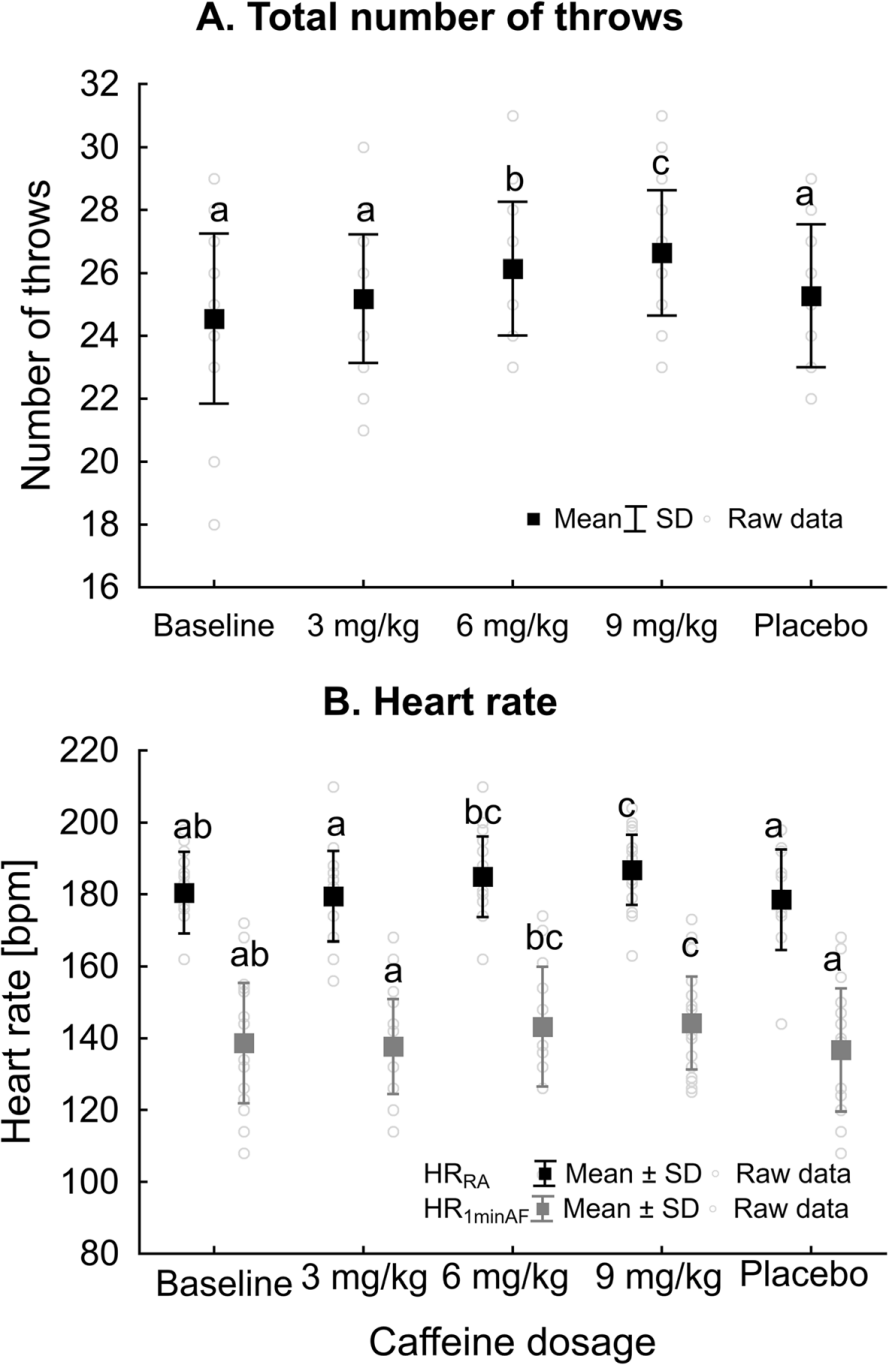
Total number of throws in SJFT (**a**) and heart rate right after (HR_RA_) and 1 min after (HR_1minAF_) the SJFT (**b**). ^abc^ - different letter inscriptions refer to statistical differences between CAF dosages; *P* <  0.05

TOT_throws_ in SJFTs were significantly improved by CAF doses (*χ*^*2*^ = 28.03, *P* <  0.001, *W* = 0.32). The 6 and 9 mg/kg doses substantially improved results compared to 3 mg/kg, PLA or baseline (BASE). Moreover, TOT_throws_ were higher at 9 mg/kg compared to 6 mg/kg (Fig. 2a). Concomitantly, HR_RA_ (*F* = 3.72, *P* = 0.008, ${\eta}_p^2$ = 0.15; Fig. 2b) and HR_1minAF_ SJTFs (*F* = 3.06, *P* = 0.02, ${\eta}_p^2$ = 0.13; Fig. 2b) were significantly increased after 6 mg/kg and 9 mg/kg CAF ingestion. 6 mg/kg CAF intake substantially increased HR_RA_ and HR_1minAF_ as compared to 3 mg/kg and PLA, and after 9 mg/kg CAF, HR measurements were additionally significantly higher compared to BASE. However, there were no differences in SJFT_Index_ between CAF dosages (Table 2).

Considering SJFT round results separately, CAF substantially improved the 1st (R_1_) and 3rd (R_3_) but not the 2nd (R_2_) round (Table 2). For SJFT_R1_, the highest number of throws was observed at 9 mg/kg; it was significantly higher compared to 3 mg/kg, PLA and BASE, but comparable to the effect of 6 mg/kg. During SJFT_R2_, 6 and 9 mg/kg CAF were more effective compared to 3 mg/kg, PLA or BASE. Overall, apart from 9 mg/kg CAF, the number of throws was significantly higher in SJFT_R2_ compared to SJFT_R3_ (Table 2).

**Fig. 3:**
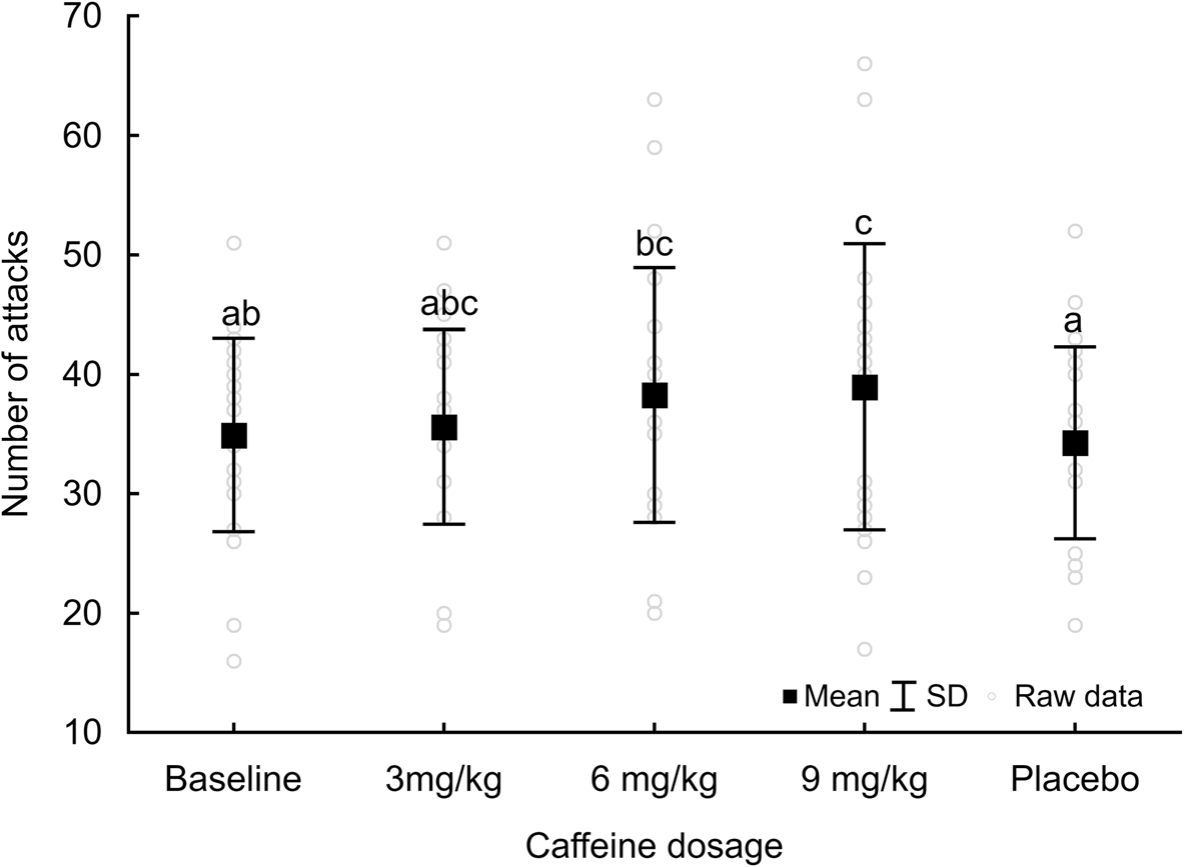
Total number of attacks for 3 consecutive combats. ^abc^ - different letter inscriptions refer to statistical differences between CAF dosages; *P* < 0.05

The total number of attacks (TOT_attacks_) in three combats were significantly improved by CAF supplementation (*F* = 2.81, *P* = 0.047, ${\eta}_p^2$ = 0.12). Nine mg/kg CAF was most effective and led to a higher number of attacks compared to BASE or PLA (Figure 3). Six mg/kg CAF was more effective than PLA but comparable to BASE. However, the effect of CAF supplementation was not observed when analysing the results of the particular combats across CAF dosages separately (Table 3). Similarly, there were no differences in number of attacks among fights 1, 2 or 3 for any tested CAF dosage or BASE and PLA (Table 3).

**Table 2:** *Special Judo Fitness Test* (SJFT) data analysis

	All	Caffeine consumers	Caffeine non-consumers	Uor t	P	r_g_ or d
	*n* = 22	*n* = 10	*n* = 12			
SJFT_R1_						
Baseline	5.3 (0.7)^a^	5.1 (0.9)^a^	5.5 (0.5)^a^	45.0	0.34	0.25
3 mg/kg	5.6 (0.6)^ab^	5.7 (0.5)^b^	5.6 (0.7)^ab^	51.5	0.60	−0.14
6 mg/kg	5.9 (0.6)^bc^	5.7 (0.5)^ab^	6.0 (0.6)^b^	45.0	0.34	0.25
9 mg/kg	6.0 (0.6)^c^	6.0 (0.5)^b^	6.0 (0.7)^b^	60.0	0.97	0.00
Placebo	5.6 (0.6)^ab^	5.6 (0.5)^ab^	5.6 (0.7)^ab^	57.0	0.87	−0.05
Χ^2^	21.46	12.85	11.67			
P	< 0.001	0.01	0.02			
Kendall’s W	0.24	0.32	0.24			
SJFT_R2_						
Baseline	10.0 (1.2)^B^	9.9 (1.3)^A^	10.1 (1.1)^B^	0.36	0.72	0.16
3 mg/kg	10.1 (1.0)^B^	9.9 (1.1)^B^	10.3 (1.0)^A^	0.98	0.34	0.42
6 mg/kg	10.4 (0.8)^B^	10.2 (0.6)^A^	10.5 (1.0)^A^	49.0	0.49	0.18
9 mg/kg	10.5 (0.8)^A^	10.7 (0.7)^A^	10.3 (0.9)^A^	41.0	0.22	−0.32
Placebo	10.3 (0.9)^B^	10.3 (0.8)^B^	10.3 (1.0)^B^	57.0	0.87	−0.05
Χ^2^ or F	6.93	6.84	0.97			
P	0.14	0.14	0.43			
Kendall’s W or ${\eta}_p^2$	0.08	0.17	0.08			
SJFT_R3_						
Baseline	9.2 (1.3)^aA^	9.1 (1.4)^A^	9.3 (1.2)^A^	0.42	0.68	0.18
3 mg/kg	9.5 (1.0)^aA^	8.9 (0.9)^A^	9.9 (0.9)^A^	26.0	0.03	0.57
6 mg/kg	9.9 (1.1)^bA^	9.6 (1.0)^A^	10.2 (1.1)^A^	43.5	0.22	0.28
9 mg/kg	10.2 (1.1)^bA^	9.9 (1.1)^A^	10.4 (1.0)^A^	42.0	0.25	0.30
Placebo	9.4 (1.1)^aA^	9.5 (1.1)^A^	9.3 (1.2)^A^	55.0	0.77	−0.08
Χ^2^	19.58	6.79	19.93	–	–	–
P	0.006	0.15	< 0.001	–	–	–
Kendall’s W	0.22	0.17	0.42	–	–	–
SJFT Index						
Baseline	13.2 (2.2)	13.9 (2.3)	12.6 (2.0)	–	–	–
3 mg/kg	12.7 (1.8)	13.5 (1.9)	12.0 (1.4)	–	–	–
6 mg/kg	12.6 (1.4)	13.1 (1.2)	12.3 (1.4)	–	–	–
9 mg/kg	12.5 (1.3)	12.7 (1.3)	12.3 (1.3)	–	–	–
Placebo	12.6 (1.7)	13.0 (1.4)	12.2 (1.9)	–	–	–
Dosage effect						
F	2.38	–	–			
P	0.06	–	–			
${\eta}_p^2$	0.10	–	–			
Dosage x caffeine consumption effect						
F	1.51	–	–			
P	0.21	–	–			
${\eta}_p^2$	0.07	–	–			
Statistical significance between SJFT_R2_ and SJFT_R3_ within the same caffeine dosage						
Z or t (Baseline)	2.56	1.56	3.00	–	–	–
P	0.01	0.15	0.01	–	–	–
r_c_ or d	0.66	0.60	0.65	–	–	–
Z or t (3 mg/kg)	2.83	2.37	1.57	–	–	–
P	0.005	0.02	0.12	–	–	–
r_c_ or d	0.79	0.89	0.64	–	–	–
Z or t(6 mg/kg)	2.13	1.52	1.83	–	–	–
P	0.03	0.14	0.07	–	–	–
r_c_ or d	0.64	0.57	0.66	–	–	–
Z or t (9 mg/kg)	1.13	1.84	−0.69	–	–	–
P	0.26	0.07	0.50	–	–	–
r_c_ or d	0.30	0.61	−0.18	–	–	–
Z or t (Placebo)	3.29	2.03	2.67	–	–	–
P	0.001	0.04	0.008	–	–	–
r_c_ or d	0.80	0.72	0.89	–	–	–

Values are means (SD). ^abc^ – different letter inscriptions refer to statistical differences between caffeine dosages; ^ABC^ – different letter inscriptions refer to statistical differences between SJFT rounds (R_2_ and R_3_) within the same caffeine dosages. SJFT, *Special Judo Fitness Test*

CAF supplementation also had no effect on the rating of perceived exertion after all the procedures (Table 4).

**Table 3:** Number of attacks during Randori combats

	FIGHT_1_	FIGHT_2_	FIGHT_3_	*F*	*P*	${\eta}_p^2$
Dosage						
Baseline	12.0 (3.9)	10.8 (3.5)	12.1 (2.6)	2.03	0.14	0.09
3 mg/kg	12.2 (3.5)	11.3 (4.1)	12.0 (4.0)	0.45	0.64	0.02
6 mg/kg	12.3 (4.7)	12.6 (3.8)	13.4 (4.4)	0.76	0.47	0.04
9 mg/kg	12.9 (4.4)	12.5 (4.1)	13.6 (4.7)	1.36	0.27	0.06
Placebo	11.5 (3.9)	11.2 (3.2)	11.5 (3.5)	0.08	0.92	0.00
Dosage effect						
*F*	0.61	1.97	1.79	–	–	–
*p*	0.66	0.11	0.14	–	–	–
${\eta}_p^2$	0.03	0.09	0.07	–	–	–
Dosage x caffeine consumption effect						
*F*	0.81	0.25	0.19	–	**–**	–
*p*	0.53	0.91	0.94	–	**–**	–
${\eta}_p^2$	0.04	0.01	0.01	–	**–**	–

Values are expressed as means (± SD)

**Fig. 4:**
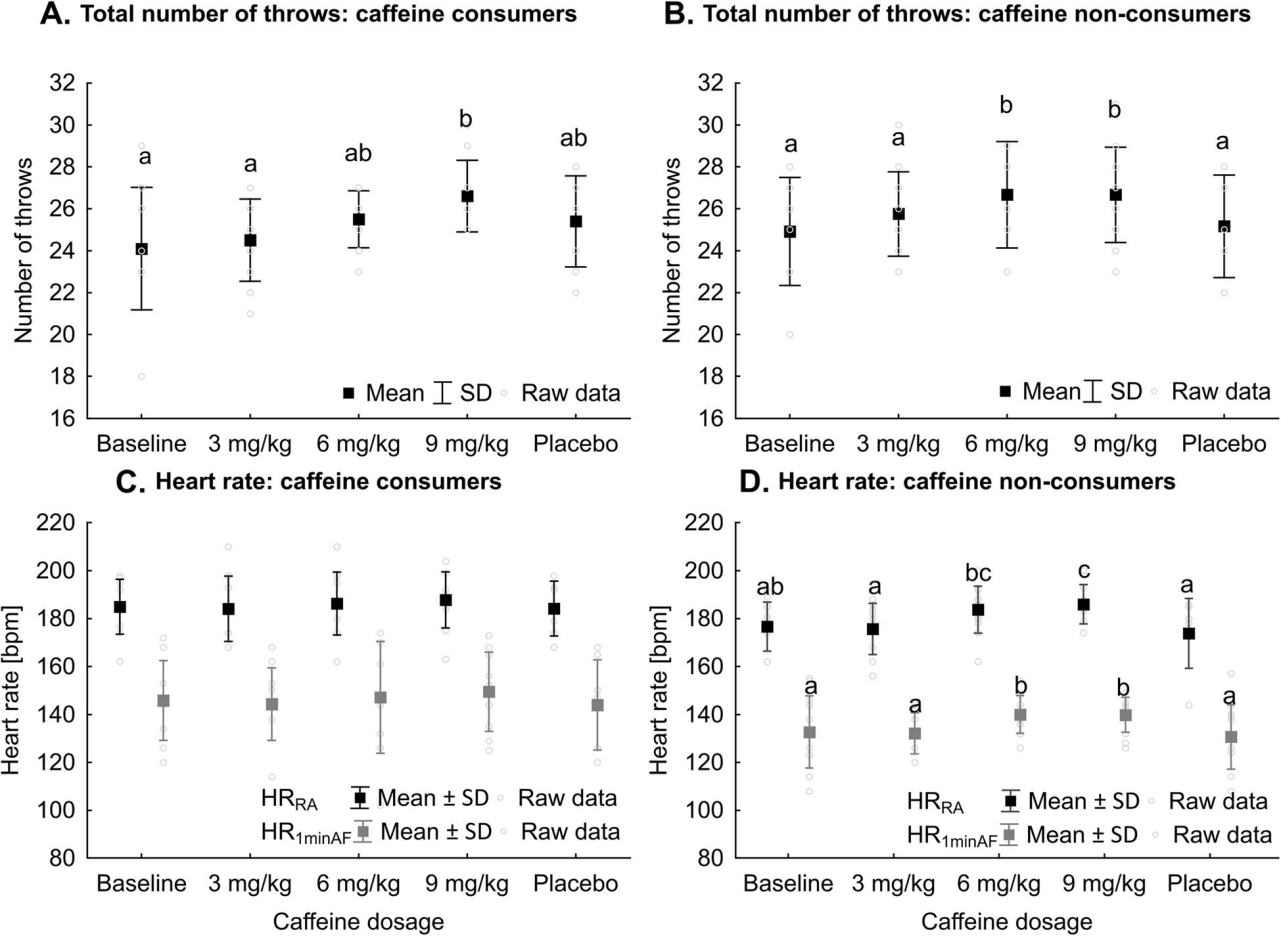
Total number of throws in SJFT (**a-b**) and heart rate right after (HR_RA_) and 1 min after (HR_1minAF_) the tests (**c-d**) in athletes who consume (**a**, **c**) or do not consume CAF (**b**, **d**) on a regular basis. ^abc^ - different letter inscriptions refer to statistical differences between CAF dosages, *P* < 0.05

In general, habitual consumption of CAF-containing products lowered the responsiveness CAF supplementation. Among those who customarily consumed CAF-containing products (*F* = 3.17, *P* = 0.02*,* ${\eta}_p^2$ = 0.12), only the 9 mg/kg dose considerably increased TOT_throws_ in SJFT compared to BASE or 3 mg/kg (Figure 4a). There were no differences between any of studied doses and PLA. Among CAF non-consumers (*F* = 7.79, *P* <  0.001*,* ${\eta}_p^2$ = 0.41), 6 and 9 mg/kg CAF doses markedly increased SJFT performance compared to BASE, 3 mg/kg or PLA (Figure 4b). In CAF consumers supplementation had no effect on HR_RA_ or HR_1minAF_ (Figure 4c). Among non-consumers 9 mg/kg CAF, compared to 3 mg/kg, BASE or PLA, considerably increased HR_RA_, and 6 mg/kg elevated HR_RA_ compared to 3 mg/kg and PLA (*F* = 3.99, *P =* 0.01*,* ${\eta}_p^2$ = 0.27,Figure 4d). Further, at 6 and 9 mg/kg CAF, HR_1minAF_ was higher compared to 3 mg/kg, BASE or PLA (*F* = 3.63, *P =* 0.01*,* ${\eta}_p^2$ = 0.25, Figure 4d). At 3 mg/kg CAF HR_1minAF_ was also higher in consumers compared to non-consumers (Table 5).

**Table 4:** Rating of perceived exertion (RPE) after all the procedures (Borg scale)

Indicator	All	Caffeine consumers	Caffeine non-consumers	*U*	*P*	*r*_*g*_
	*n* = 22	*n* = 10	*n* = 12			
RPE						
Baseline	13.8 (2.2)	14.3 (2.6)	13.4 (1.8)	45.00	0.34	−0.25
3 mg/kg	14.2 (2.3)	14.5 (1.4)	13.9 (2.9)	50.50	0.55	−0.16
6 mg/kg	13.6 (1.7)	13.7 (1.4)	13.5 (2.0)	59.50	1.00	0.01
9 mg/kg	13.5 (1.3)	13.9 (1.3)	13.2 (1.3)	46.00	0.35	−0.23
Placebo	13.0 (2.6)	13.6 (2.5)	12.6 (2.6)	47.00	0.41	−0.22
*Χ*^*2*^	3.22	3.16	1.46	–	–	–
*P*	0.52	0.53	0.83	–	–	–
Kendall’s *W*	0.04	0.08	0.03	–	–	–

Values are expressed as means (± SD). RPE, Rate of Perceived Exertion

Considering results of SJFT rounds separately, for both CAF consumers and non-consumers, the ergogenic effect of CAF supplementation was observed only at SJFT_R1_ (Table 2). Among CAF consumers, the 3 and 9 mg/kg doses increased the number of throws compared to BASE. None of the doses where more effective than PLA. Among non-consumers the 6 and 9 mg/kg doses increased the number of throws compared to BASE, yet not to PLA. At 3 mg/kg CAF, the SJFT_R3_ result was higher in non-consumers compared to consumers. In CAF consumers the number of throws at 3 mg/kg and PLA was significantly higher in SJFT_R2_ compared to SJFT_R3._ In non-consumers the corresponding differences were observed at BASE and PLA (Table 2). Further, TOT_attacks_ (*F* = 0.04, *P* = 0.98*,* ${\eta}_p^2$ = 0.002) in each separate combat was not affected by customary consumption of CAF-containing products, as determined by two-way ANOVA with RM (Table 3).

**Table 5:** Summary of comparisons between caffeine consumers and non-consumers among study participants in SJFT

Indicator	Baseline	3 mg/kg	6 mg/kg *n* = 22	9 mg/kg	Placebo
Total number of throws in SJFT					
*t*	0.70	1.47	1.31	0.08	−0.23
*P*	0.49	0.16	0.21	0.94	0.82
*d*	0.30	0.63	0.56	0.03	−0.10
HR immediately after SJFT (HR_RA_)					
*t*	−1.80	−1.63	−0.52	−0.42	−1.83
*P*	0.09	0.12	0.61	0.68	0.08
*d*	−0.77	−0.70	−0.22	− 0.18	−0.78
HR 1 min after SJFT (HR_1minAF_)					
t^*^	−1.94	−2.36	−0.99	−1.84	− 1.93
*P*	0.07	0.03	0.33	0.08	0.07
*d*	−0.83	−1.01	− 0.43	− 0.79	−0.83

Values are t-score (t), *P*-value and Cohen’s d. SJFT, Special Judo Fitness Test; HR, heart rate

## Discussion

This study is the first to investigate the effect of acute variable CAF supplementation compared to PLA on discipline-specific performance and combat activity in judo. Our study results support the hypothesis that the ergogenic effect of CAF is dose-dependent in the range of the studied dosages, and that regular customary consumption of CAF-containing products alters the effect of CAF supplementation. The main finding is that, in general, 6 and 9 mg/kg CAF improved TOT_throws_ in SJFT compared to 3 mg/kg, PLA or BASE. Importantly, the commonly used 3 mg/kg dosage did not substantially improve performance compared to PLA or BASE. Nine mg/kg CAF exclusively increased combat activity compared to PLA or BASE. Among CAF non-consumers 6 mg/kg was as effective as 9 mg/kg in enhancing SJFT performance. Among judoists who habitually consumed CAF-containing products, only 9 mg/kg CAF was more effective than 3 mg/kg or BASE. Thus, we conclude that with regard to combat sports, higher (6–9 mg/kg) than currently recommended CAF dosages (3–6 mg/kg) [1, 2, 5] are apparently more effective in terms of discipline-specific performance.

CAF easily crosses the blood-brain barrier and acts in the CNS [13, 15]. It also crosses cellular membranes of other tissues [9], thus many mechanisms explaining its ergogenic action were uncovered [3, 5, 7–10, 37–39]. Possible CAF mechanisms of action include: competition with adenosine receptors [8, 13, 14]; an increase in calcium ion release from the sarcoplasmic reticulum [13, 15]; norepinephrine elevation [4, 20, 21] and increasing HR [4, 8, 40, 41]; stimulates beta-endorphin secretion and decreasing pain perception [42] or altering skeletal and neuromuscular functions [15, 43, 44] and promoting a thermogenic response [45]; interference in substrate utilisation during exercise, by decreasing reliance on glycogen utilisation and increasing fat oxidation [10]. CAF supplementation purportedly increases time to exhaustion [10, 46, 47], modulates central fatigue [37], reduces RPE [38], improves agility and decision making [4, 11, 12, 19] and increases alertness and cognitive performance [8, 17, 18]. CAF exerts significant ergogenic effects on strength and power; nevertheless, there is a need for future studies that explore the optimal CAF form and dosage to maximise its effect on muscles [48].

Combat sports require high levels of power, strength, dynamics and agility [4]. CAF effects in high-intensity intermittent combat sport exercises are not highly recognised. It is supposed that in combat sports CAF may indirectly contribute to an increase in blood lactate concentrations after specific tests and simulated combats [25, 44, 49]. This effect is probably due to CNS stimulation [23, 44], higher energy availability for muscles [44] and/or reduced pain perception [23, 44]. All of the mentioned mechanisms may lead to greater ability to exercise with higher intensity and to a higher volume of performed effort. These could be the direct reasons for increase in blood lactate concentration. It is also possible that CAF enhances muscle contractions, increases technical performance and/or delays fatigue during combats [44].

### SJFT

A few studies examined the effects of CAF on performance in combat sports. Yet, there are no trials that compared different supplementation strategies (doses) in judo. One previous study in endurance athletes suggested that significant performance increases can only be achieved by low to moderate CAF doses (3–6 mg/kg), while high doses (9 mg/kg) may overstimulate the CNS [50]. In the current study, we showed that CAF doses of 6 and 9 mg/kg resulted in better SJFT performance compared to BASE, PLA or 3 mg/kg. Further, at 9 mg/kg CAF the number of throws between SJFT_R3_ and SJFT_R2_ was comparable. Astley et al. [29] noted that acute ingestion of 4 mg/kg CAF increases the number of throws in SJFT compared to PLA in young judoists, while Lopes-Silva et al. [25] and Felippe et al. [51] did not observe any effects of 6 mg/kg CAF alone on the number of throws. Inconsistences between findings in the aforementioned studies may arise from different methodological approaches, e.g., different athlete weight categories, alternative pre-supplementation preparation (e.g., 5-day weight loss weight [25]) or small sample sizes [25, 51].

Other studies that investigated the effects of 5 mg/kg of CAF supplementation on judo performance [44, 52] utilised measures we did not implement, namely the Wingate Anaerobic Test [52], countermovement jump test, handgrip strength test or judo grip strength test [44]. Athayde et al. [44] did not observe any positive significant effect of CAF supplementation in neuromuscular tests. Souissi et al. [52], however, noted that CAF ingestion improves peak and mean power after morning compared to afternoon supplementation. CAF effectiveness was also investigated in wrestling [53, 54], taekwondo [55, 56], Brazilian jiu-jitsu (BJJ) [49, 57] and boxing [58]. Four [54] and 5 mg/kg [53] doses were ineffective; only 10 mg/kg CAF [54] improves performance in wrestlers. Further, supplementation with 5 mg/kg CAF produced mixed results in taekwondo athletes [55, 56]; 3 mg/kg enhances combat intensity and muscular performance in BJJ [49, 57] and 6 mg/kg induces greater duration of high-intensity actions in boxing [58].

### Combat activity

Judo combat is dependent on the opponent’s strategy of attacks and defense, and it is composed of acyclic tasks, factors which make it difficult to identify the effect of CAF during combats. However, high-intensity efforts lead to central and muscle fatigue (related to metabolite accumulation, energy substrate depletion, neuromuscular junction failure and muscle contractile potential disturbance) [59]. These phenomena apparently explain why during consecutive matches the number of attacks decreases [44]. However, in our study we did not observe substantial differences in the number of attacks in three consecutive judo combats at any CAF dose, PLA or BASE. There is only one previous study [44] that measured the number of attacks during a judo match. Ingesting 5 mg/kg CAF did not increase the number of attacks [44]. This finding is contrary to our results, where we observed increased TOT_attacks_ in 3 combats at 6 and 9 mg/kg CAF comparing to PLA.

### HR

Ingesting CAF can influence both HR at rest [8, 40] and during exercise [4, 41], namely due to CNS activation (e.g., catecholamine release), phosphodiesterase inhibition, adrenal cortex stimulation (corticosteroid release) and influence on the renal system [60]. Low CAF doses (3 mg/kg) do not alter HR at rest [29, 50]. In our study 6 and 9 mg/kg CAF increased HR_RA_ and HR_1minAF_ compared to 3 mg/kg, and 9 mg/kg additionally compared to PLA. However, 3 mg/kg CAF did not affect HR, and this result is in accordance with the current literature [29, 50]. In contrast, two former studies that measured HR in judo did not observe any significant differences in HR after 4 [29] or 6 mg/kg [25] CAF supplementation.

### RPE

CAF ingestion may reduce RPE, which could be caused by the ability of CAF to cross the blood-brain barrier and compete with adenosine receptors [8, 13, 14]. This mechanism is also responsible for reduced pain perception and sleepiness [25]. On the one hand, our current study did not show any direct significant effect of CAF supplementation on RPE measured by the Borg scale and are in line with a study by Fellipe et al. [51], who utilised 6 mg/kg CAF. On the other hand, it should be emphasized, that in the current study 6 and 9 mg/kg CAF doses improved judo-specific performance. An increase in performance is inherent with, among others, greater work performed. In practice, the participants were able to perform more effectively without perceiving greater exertion. Thus, it must be stated that CAF supplementation positively affected RPE. Contrarily, report by Lopes-Silva et al. [25] showed reduced RPE in judoists who ingested 6 mg/kg CAF, but no increase in performance.

### Habitual caffeine consumption

It is important to note that, to our knowledge, there is no data on the influence of customary CAF consumption on the effectiveness of CAF supplementation in combat sports. This aspect was investigated in athletes who represented other sport disciplines [20, 61, 62]. Moreover, none of the studies showed significant differences in performance between CAF users and nonusers [20, 61, 62]. However, in CAF non-consumers, the post-ingestion effect lasted 3 h longer compared to customary consumers [61]. In our study we showed that CAF consumers needed to ingest 9 mg/kg to achieve better SJFT results, while in non-consumers the dose of 6 mg/kg was equally effective as 9 mg/kg. Further, in CAF consumers, in contrast to non-consumers, the implemented supplementation did not affect HR after exercise. Habitual CAF consumption also had no effect of combat activity.

### Implications for performance

Knowledge of the impact of different CAF dosages on judo performance could have significant beneficial implications for athletes who compete in international matches. Our data can therefore be translated into a real-world setting and hold implications for improving performance and overall competition efficiency. Information on whether higher CAF doses promote more throws and/or attacks could be important to establish discipline-dependent recommendations for CAF usage. Importantly, given the physiologically similar nature of the effort, our results could also be used also in ergogenic supplementation support of athletes in wrestling, BJJ, and other “grappling” combat sports disciplines.

### Limitations and strengths

It should be noted that our research has some limitations. First, we did not analyse blood biochemical marker (e.g., CAF) concentration in athletes during our study, which in our opinion would be worth performing in future investigations. However, our primary outcome was a practical assessment of the final CAF influences on discipline-specific exercise capacity, which we achieved. Second, the result of combat activity may be also related to the opponent’s experience level and the load that the opponent makes during the combat. Still, in our opinion this is in practice an unavoidable problem in combat sports. However, to minimise this effect, judoists fighting with each other were selected in terms of weight category and experience level, as well as in the same order of opponents (in T_1_-T_5_). Further, the result of combat activity may be affected by the fatigue that occurred after SJFT. According to RPE expressed by the Borg scale [30, 63], the participants perceived the exertion after SJFT as somewhat hard. Finally, we did not account for carry-over effect. However, it needs to be emphasised that at each laboratory visit we administered a single CAF dose to each participant, and each participant visited the laboratory five times with at least 7 days of wash-out. Since the elimination half-life of CAF ranges between 2.5 and 10 h [3], we believe that this wash-out period was sufficient to remove CAF from the judoist’s systems.

The unquestionable strength of our study is application of a four-fold crossover design protocol and implementation in all study participants each of the three CAF doses as well as PLA; such an approach has never been deployed in judo before. For the scientific value of our work, double-blind masking and full control of compliance of the delivery of the administered supplement, which was taken at the set time under the control of a member of the research team, are also important. Moreover, there was close cooperation among the research team and the coaches to ensure proper study protocol conduct (e.g., motivated athlete effort and commitment and analysed and assessed the correctness of techniques and actual attacks during combats). It is also worth noting that we used the discipline-specific judo performance test. The SJFT was developed to evaluate both anaerobic and aerobic potential, and it can be considered more appropriate to evaluate judo performance capacity than the Wingate Anaerobic Test [31–33]. It is also worth taking into account the high training level of studied judoists and high familiarisation of the studied group to perform all efforts. We also accounted for customary consumption of CAF-containing products. We revealed that recommended doses of CAF in judo should vary according to usual caffeine consumption.

## Conclusion

Our study indicated that acute pre-exercise CAF supplementation effectively supports performance and exercise capacity in judo. Six and 9 mg/kg CAF doses improved discipline-specific performance, while 9 mg/kg increased combat activity. The 3 mg/kg CAF dose did not have any positive effect. The ergogenic effect of CAF is not only dose-dependent but also related to customary CAF-consumption. Among CAF non-consumers, 6 mg/kg CAF was equally efficient to 9 mg/kg in enhancing performance, while in habitual CAF consumers, only 9 mg/kg was more effective compared to 3 mg/kg or PLA. On the basis of our results, we conclude that with regard to combat sports, higher (6–9 mg/kg) than currently recommended CAF dosages (3–6 mg/kg) are apparently more effective in terms of specific judo performance.

## Additional file

Additional file 1: **Table S1.** CONSORT checklist. (PDF 145 kb)

## Data Availability

The datasets used and/or analysed during the current study are available from the corresponding author on request.

## Abbreviations

BASE: Baseline
BJJ: Brazilian jiu-jitsu
CAF: Caffeine
CNS: Central nervous system
HR: Heart rate
PLA: Placebo
RPE: Rating of perceived exertion
SJFT: *Special Judo Fitness Test*
